# Identification of thrombotic biomarkers in orthopedic surgery patients by plasma proteomics

**DOI:** 10.1186/s13018-023-03672-1

**Published:** 2023-03-21

**Authors:** Cui-Qing Liu, Yu-Jing Gao, Geng-Xiong Lin, Jun-Ze Liang, Yan-Fei Li, Yi-Chun Wang, Wen-Yan Chen, Wei-Ju Chen

**Affiliations:** 1 School of Nursing, Jinan University, Guangzhou, 510613 China; 2 The First Affiliated Hospital, Jinan University, Guangzhou, 510632 China; 3 College of Life Science and Technology, Jinan University, Guangzhou , China

**Keywords:** Proteomics, Venous thromboembolism, Data-independent acquisition mass spectrometry, Orthopedic surgery, Von Willebrand factor

## Abstract

**Background:**

Due to the poor specificity of D-dimer, more accurate thrombus biomarkers are clinically needed to improve the diagnostic power of VTE.

**Methods:**

The plasma samples were classified into low-risk group (*n* = 6) and high-risk group (*n* = 6) according to the Caprini Thrombosis Risk Assessment Scale score. Data-independent acquisition mass spectrometry (DIA-MS) was performed to identify the proteins in the 12 plasma samples. Bioinformatics analysis including volcano plot, heatmap, KEGG pathways and chord diagram analysis were drawn to analyze the significantly differentially expressed proteins (DEPs) between the two groups. Then, another 26 plasma samples were collected to verify the key proteins as potential biomarkers of VTE in orthopedic surgery patients.

**Results:**

A total of 371 proteins were identified by DIA-MS in 12 plasma samples. Volcano plotting showed that there were 30 DEPs. KEGG pathway enrichment analysis revealed that the DEPs were majorly involved in the blood coagulation pathway. The chord diagram analysis demonstrated that proteins SAA1, VWF, FLNA, ACTB, VINC, F13B, F13A and IPSP in the DEPs were significantly related to blood coagulation. VWF and F13B were selected for validation experiments. ELISA test showed that, as compared with those in the low-risk group, the level of VWF in the high-risk sera was significantly increased.

**Conclusions:**

The level of VWF in the high-risk group of thrombosis after orthopedic surgery was significantly higher than that in the low-risk group of preoperative thrombosis, suggesting that VWF may be used as a potential thrombus biomarker in orthopedic surgery patients.

**Supplementary Information:**

The online version contains supplementary material available at 10.1186/s13018-023-03672-1.

## Introduction

Venous thromboembolism (VTE) is the leading reason of cardiovascular death worldwide. The annual incidence of VTE was estimated to be approximately 1–2 per 1000 population in Europe and the USA, and the incidence increases with age growth [1–3]. Approximately two-thirds of patients with symptomatic VTE manifest deep vein thrombosis alone (DVT), while one third has pulmonary embolism (PE) [3–5]. According to Virchow triad, the main factors for the formation of thrombus include stasis of blood flow, endothelial injury and hypercoagulability [6]. In orthopedic patients, bone fracture can lead to venous injury. Immobilization and bed rest after surgery cause blood stasis in the lower extremities of the patients. In addition, fasting, anesthesia, hemostatic drugs, intraoperative blood loss and postoperative fever lead to the patients' blood hypercoagulability. These factors result in high incidence of VTE in patients after orthopedic surgery. More than 10% incidence of VTE has been reported after knee or hip joint replacement surgery [7]. And PE contributes to a mortality rate of 70% among patients [8]. Unfortunately, there may be no obvious signs of thrombosis, and thrombosis can occur at any age and at any time, seriously threatening the life and health of patients. Therefore, early diagnosis and prevention of thrombosis are crucial.

Currently, evaluating whether a patient suffers from VTE is mainly based on the patient's symptoms, signs, and auxiliary examinations. Computed tomography pulmonary angiography (CTPA) has been applied as the gold standard for diagnosing PE. However, radiation exposure and expensive price restrict the application of the diagnosis [9]. Featuring with high sensitivity and accuracy, color Doppler ultrasound has been applied to the diagnosis of DVT [10, 11]. However, the guiding value for clinical doctors to prevent thrombosis is limited. As a final degradation product of cross-linked fibrin, D-dimer is often elevated in patients who are suffering from acute VTE [12]. D-dimer is a biomarker of coagulation activation and secondary hyperfibrinolysis, and its elevated value does not necessarily indicate thrombosis [13]. Obviously, the specificity of D-dimer for the detection of VTE is poor [14, 15]. Recent studies have attempted to identify new biomarkers that can act as a complement to D-dimer [16, 17]. However, the value of these biomarkers is susceptible to many factors such as acute coronary syndromes, local or systemic infection, trauma and malignancy [18]. Therefore, there is a need for more accurate biomarkers for predicting VTE.

Proteomic technology is an efficient tool for identifying potential biomarkers associated with pathological states [19, 20]. With the rapid development of mass spectrometry (MS), proteomics can be used to accurately profile global proteomic alterations in various cells, tissues and body fluids. By applying proteomic techniques to serology, more potential clinical plasma biomarkers associated with venous thromboembolism will likely be discovered. With high reproducibility and sensitivity, data-independent acquisition mass spectrometry (DIA-MS) [21] can generate digital proteomic maps, providing highly reproducible retrospective analysis on cell and tissue samples and thus holding great promise for biomarker discovery.

In this work, DIA-MS proteomics was performed to quantify and analyze the expression of proteins in 12 plasma samples that were classified into two groups with low and high risk of VTE according to Caprini score. Bioinformatic tools including volcano plot, heatmap, KEGG pathways enrichment and chord diagram analysis were employed to sort out the key proteins involved in related pathways associated with thrombus. Then, ELISA experiment was applied to another 26 plasma samples to verify the key proteins as potential biomarkers of VTE in orthopedic surgery patients.

## Materials and methods

### Study subjects

**Fig. 1:**
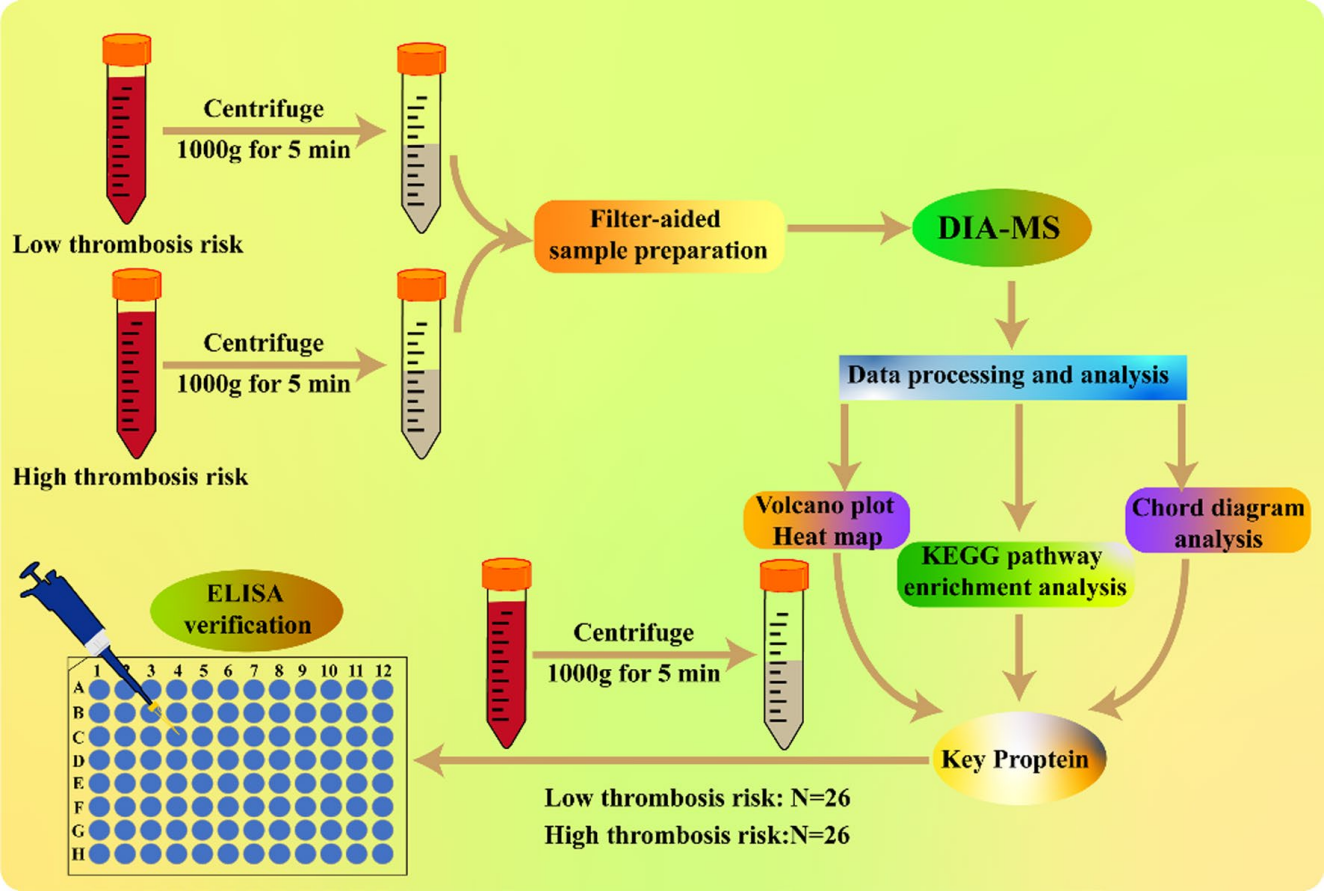
Workflow of DIA-MS analysis on plasma samples from orthopedic surgery patients with different thrombosis risk

All plasma samples came from the patients who were admitted to our department from January 2019 to January 2021. The basic characteristics of these patients were collected. And they were classified into two groups according to Caprini Thrombosis Risk Assessment Scale [22]. A total score of 0 to 1 was defined as low risk of thrombosis, 2 as intermediate, 3 to 4 as high and ≥ 5 as very high. All the work described in this manuscript has been carried out in accordance with the Code of Ethics of the World Medical Association. Approval for this study was obtained from the institutional review board of our institution. All samples from patients were analyzed under the same procedures (Fig. 1).

### Preparation of samples for DIA-MS analysis

Blood samples were collected into 5-ml anticoagulant tubes with ethylenediaminetetraacetic acid (EDTA), and, immediately, all sample tubes were homogenized upside down. Afterward, all blood specimens collected from the patients were centrifuged at 1000 g for 5 min at 4 °C, and then the respective supernatants were collected to a new corresponding centrifuge tube, which were stored at − 80 °C before further processing. Before DIA-MS analysis, the frozen plasma samples were thawed at 4 °C. The concentration of the plasma protein was detected by BCA protein assay kit (Beyotime, P0012S). Urea buffer (8 M, 500 µL) was added to 500 μL plasma samples, and the mixture was vortexed for 30 s; then, each sample was added with DTT (50 mM) and fully vortexed. After 1 h in 37 °C, IAA was added in the samples and stood for 30 min at room temperature in a dark environment. The above mixed solution was then filtered with a flat-bottom ultrafiltration tube. The filtrate was collected in a new centrifuge tube. TEAB (50 mM) and trypsin (1 μg/μL) were orderly added in the filtrate. After 16–18 h in 37 °C, the samples were centrifuged at 12,000 g for 15 min. Finally, freeze-dried protein powder was obtained in a freeze dryer. All the freeze-dried protein powders were stored at − 80 °C. For DIA-MS analysis, the protein powders were dissolved in appropriate gold water containing 0.1% FA to get the samples with the concentration of 0.5 μg/μL. Then the samples were centrifuged twice at 12,000 g for 10 min each time, and the supernatants were transferred to a new centrifuge tube. The labeled peptide (5 × iRT) was added to the centrifuge tube. Then the tubes were fully vortexed and centrifuged twice at 12,000 g for 10 min. The supernatants were collected and applied to DIA-MS analysis.

### DIA-MS conditions

The collected supernatants in “Preparation of samples for DIA-MS analysis” secttion were taken for DIA-MS analysis. All samples were separated by a nano-flow HPLC liquid phase system Easy nLC2000. Liquid phase parameters: (a) column information: 300 µm idx 5 mm, Acclaim PepMap RSLC C18, 5 µm, 100 Å (Thermo, 160,454); Acclaim PepMap 75 µm × 150 mm C18, 3 µm, 100 Å (Thermo, 160,321). (b) Mobile phase information: Mobile phase A: 0.1% formic acid. Mobile phase B: 0.1% formic acid, 80% ACN. Flow rate = 300 nL/min. (c) Analysis time = 65 min. Effective gradient: B phase rose from 5 to 90%; 0–5 min: the linear gradient of liquid B was 0–5%; 5–45 min: the linear gradient of liquid B was 5–50%; 45–50 min, the linear gradient of liquid B was 50–90%; 50–55 min: the linear gradient of liquid B maintained at 90%; 55–65 min: the linear gradient of liquid B decreased to 5%. Mass spectrometry parameters were as follows: (a) Primary mass spectrometry parameters: resolution = 70,000; AGC target = 3e6; maximum IT = 40 ms; scan range = 350 to 1800 m/z. (b) MS parameters: resolution = 17,500; AGC target = 1e5; maximum IT = 60 ms; Top *N* = 20; NCE/stepped NCE = 27.

### Data processing and analysis

The original data were transformed into.MzML format by Trans-Proteomic Pipeline software which also was used for database search. The theoretical protein sequence database is the Uniport human nonredundant library.fasta format file; the search database used the comet search engine and the corresponding file.params format; no decoy was added, the whole enzyme was used, and the trypsin missed cleavage site was set to 0. The fixed modified cysteine was alkylated (57.02146 Da), and the non-fixed modified methionine was oxidized (15.9949 Da). The error range of precursor ions was controlled within 10^–5^, and the error of secondary debris was controlled within 0.02 Da. The 12 database search results were integrated and constructed by TPP and imported into Skyline for data analysis of DIA.

DIA data acquisition was performed on 12 samples, and the mass scanning range m/z 400–1200 was equally divided into 32 consecutive 25 u windows. In each window, all product ion information of all precursor ions was selected and repeated. The Skyline software was used for quantitative analysis of label-free proteomics. DIA data were imported to screen and extract protein quantitative information. The automatic auxiliary function of Skyline was used to screen suitable chromatographic peaks. Information on protein name, peptide sequence, and product ion area was exported.

Two DIA quantification data were normalized by total ionic strength (TIC) via R (version 3.2.2) software, and the average of the two results was used for subsequent analysis. The peptides with ion quantitative correlation of more than 0.6 in the two groups of samples were retained. The product ion areas of the same peptides were summed to the peptide level, and two-tailed t-test was performed at the peptide level to screen peptides with significant difference (*P* < 0.05). The different peptides were normalized to the protein level to get DEPs. Then, the R language was applied to draft the volcano plot for visualizing the expression of proteins in plasma and Heatmap for cluster analysis. The data of DEPs were uploaded to the KEGG database (http://www.genome.jp/kegg/) for enrichment analysis of metabolic pathways. Chordogram analysis of GO enrichment was performed to present the DEPs in three pathways of blood coagulation, humoral immune response and acute phase response.

### ELISA

Another 26 plasma samples of each group were collected from patients using the method described in “Preparation of samples for DIA-MS analysis” section. Levels of VWF and F13B in the plasma samples were measured using ELISA kit according to the instruction manual.

### Statistical analysis

All date were imported into GraphPad prism 8.3.0 for analysis. All results of ELISA were imported into SPSS 22.0 software (IBM, Armonk, NY, USA) for statistical analysis. Measurement data are presented as mean ± standard deviation (SD). One-way ANOVA was used for statistical analysis. A value of *P* < 0.05 was considered statistically difference.

## Results

### Patient baseline characteristics

The basic information of the 6 patients is shown in Table 1. The information of age, gender, height, weight, BMI, hypertension, diabetes, diagnosis and Carprini score are presented in the table. Apart from the factor of orthopedic surgery, there is no any difference between the samples of two groups.

**Table 1:** Basic information of 6 orthopedic patients

Patient number	Age (Y)	Gender (male/female)	Height (cm)	Weight (kg)	BMI (kg/m^2^)	Hypertension (Yes/No)	Diabetes (Yes/No)	Diagnosis	Preoperative Carprini score (low risk)	Postoperative Carprini score (high risk)
1	27	Male	182.0	82.0	24.6	Yes	No	LDH	0	7
2	55	Male	169.0	78.2	27.3	Yes	No	LDH	1	8
3	57	Male	168.0	76.6	27.0	Yes	No	LSS	1	6
4	49	Female	155.8	64.3	26.5	No	No	OVCF	1	9
5	14	Male	162.0	42.0	16.0	No	No	LDH	0	8
6	51	Female	167.0	76.5	27.4	Yes	No	OVCF	1	7

*LDH* Lumbar Disc herniation, *LSS* Lumbar spinal stenosis, *OVCF* Osteoporotic vertebral compression fracture

### The results of DIA-MS

**Fig. 2:**
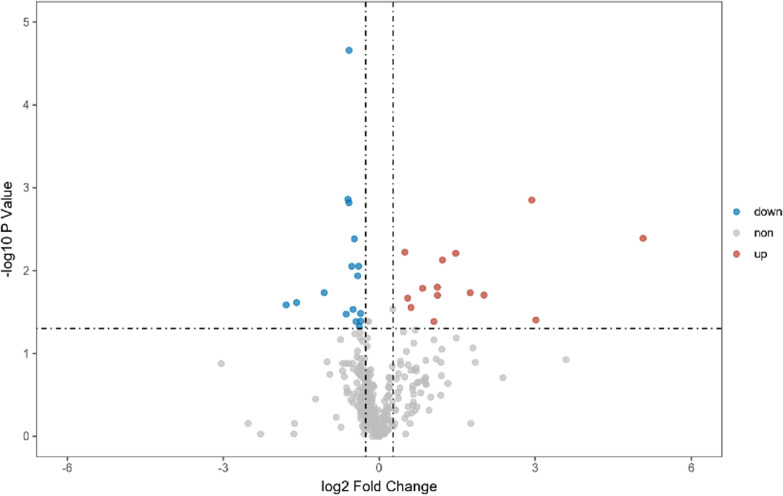
Volcano plot of differentially expressed proteins (DEPs) in sera from patients at high and low risk of thrombosis. Fold change > 2, *P* < 0.05 means that the protein expression is upregulated, indicated by red dots; fold change < -− 2, *P* < 0.05 means that the protein expression is downregulated, indicated by blue dots

**Fig. 3:**
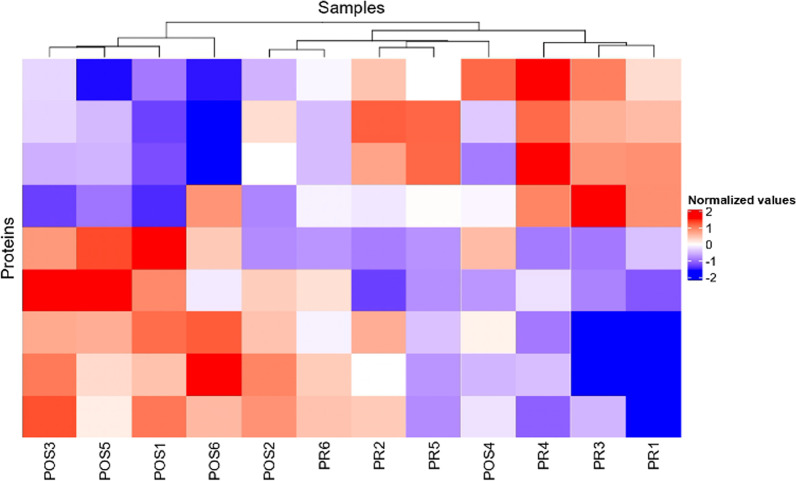
Heatmap for cluster analysis of DEP genes between the two groups. Each column represents a sample, and the sample number is shown below the corresponding column; each row represents a differentially expressed gene. Red indicates that the differential gene expression was upregulated compared with another group (*P* < 0.05). Blue indicates that the differential gene expression was downregulated compared with another group (*P* < 0.05)

A total of 371 proteins were identified in 12 plasma samples and 30 DEPs were found between the two sample groups before and after orthopedic surgery. The volcano plot (Fig. 2) and the heat map analysis (Fig. 3) of the 30 DEPs showed that the expression of 14 proteins was upregulated and 16 proteins were downregulated in the high-risk group of thrombosis as compared with the low-risk group of thrombosis.

**Fig. 4:**
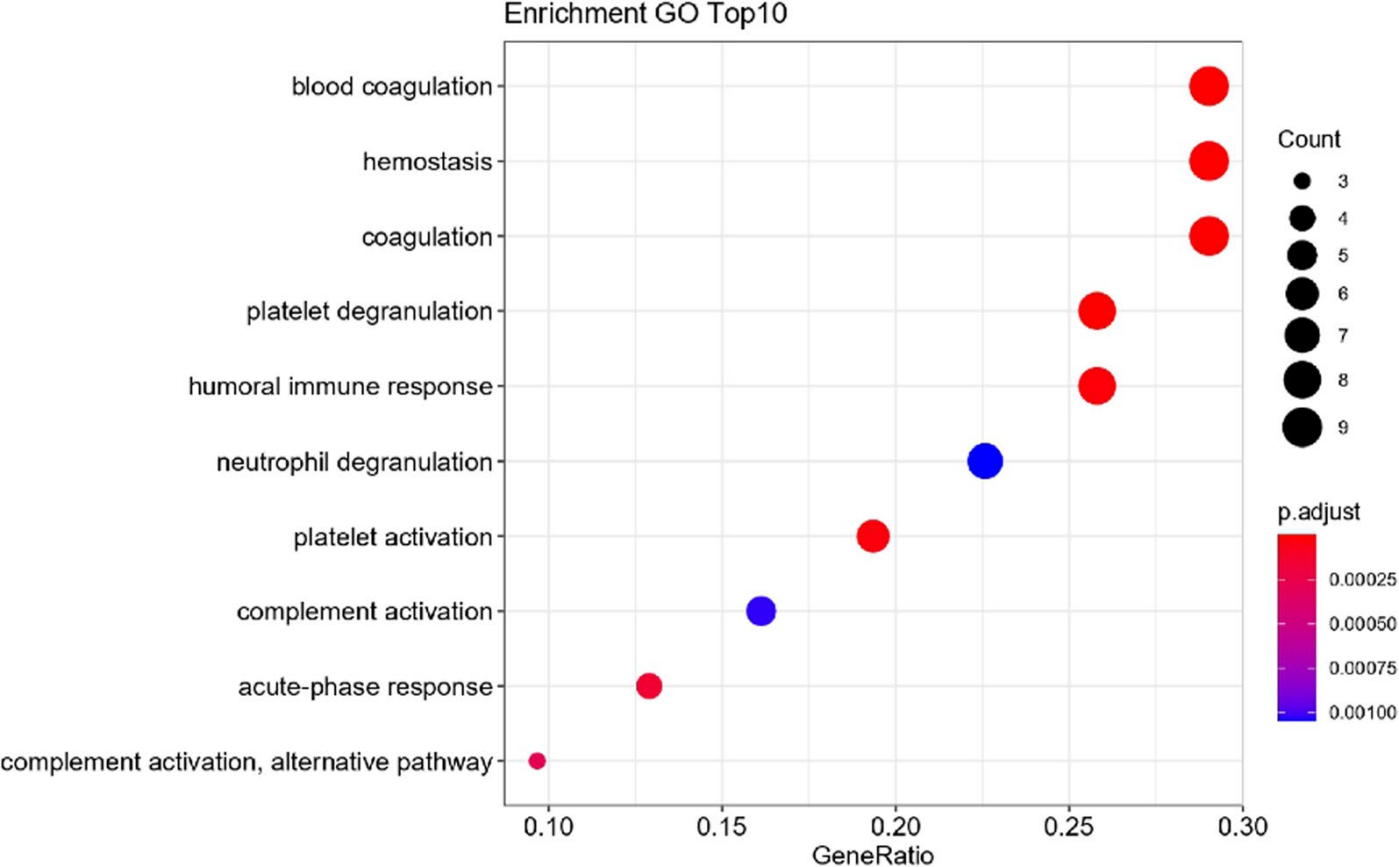
Enrichment analysis of KEGG pathways DEPs. The ordinate is the name of the KEGG pathway, and the abscissa is the enrichment fold of the pathway. The number of differentially expressed genes is represented by the size of the bubble, and the *P* value is represented by color. The top ten pathways are: blood coagulation, hemostasis, coagulation, platelet degranulation, humoral immune response, neutrophil degranulation, platelet activation, complement activation, acute phase response and alternative pathway of complement activation

Next, we performed KEGG pathway enrichment analysis on the DEPs. The results showed that the top ten pathways participated by the DEPs were (listed in descending order), blood coagulation, hemostasis, coagulation, platelet degranulation, humoral immune response, neutrophils Cell degranulation, platelet activation, complement activation, acute phase response, and alternative pathways of complement activation (Fig. 4). It can be seen that blood coagulation pathway was mainly involved.

**Fig. 5:**
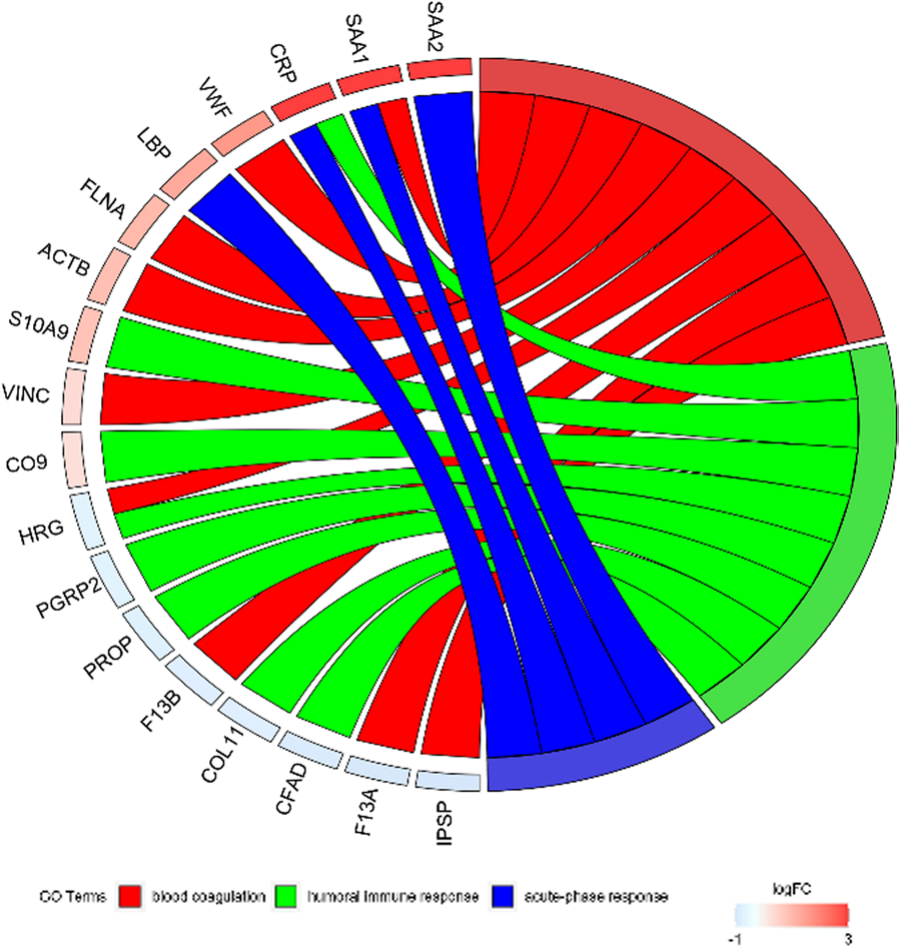
Chordogram analysis of GO enrichment of DEPs. The enrichment of DEPs in three related pathways of blood coagulation, humoral immune response and acute phase response. The left side shows DEPs, and the color indicates the degree of difference. The red chords represent the pathway related to blood coagulation, the green chords represent the humoral immune response pathway, and the blue chords represent the acute phase response pathway

Then, we performed chord diagram analysis on the DEPs, by focusing on three relevant pathways of blood coagulation, humoral immune response and acute phase response. We found that the proteins SAA1, VWF, FLNA, ACTB, VINC, F13B, F13A and IPSP were clearly associated with blood coagulation (Fig. 5). Among them, the expressions of VWF and SAA1 were significantly upregulated, while the expressions of F13B, F13A and IPSP were significantly downregulated.

### The results of ELISA

**Fig. 6:**
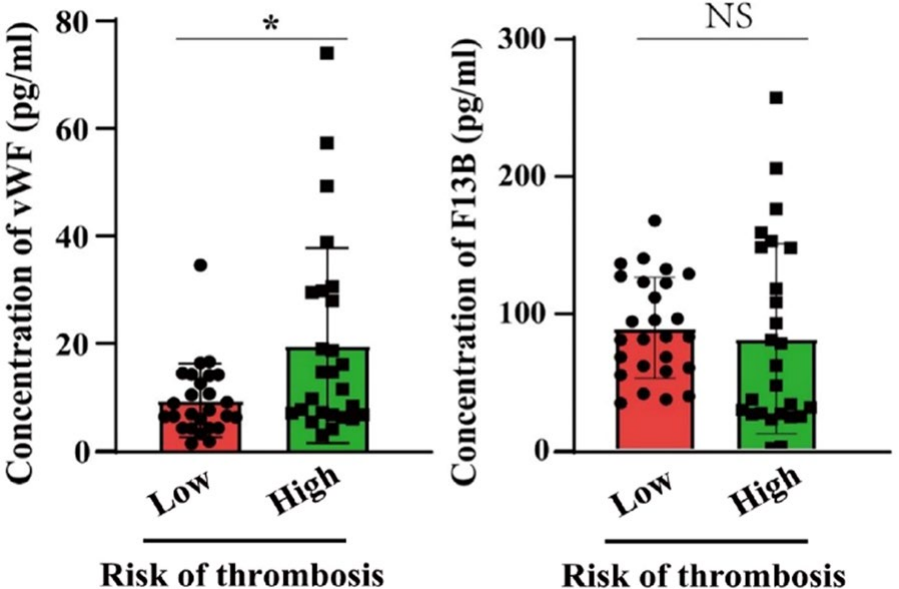
VWF protein as a potential protein biomarker for thrombotic risk in orthopedic surgery patients. Comparison of the concentrations of VWF (left panel) and F13B (right panel) in sera of different coagulation risk groups in orthopedic surgery, * means *P* < 0.05, NS means no statistical difference

We then performed ELISA experiments to validate the results obtained from our proteomic analysis. Another 26 pairs of plasma samples were thus collected in our department (Additional file 1: Tables S1, S2) for this validation experiments. ELISA testing was carried out on each sample to measure the concentration of VWF and F13B, respectively, selected from the up- and downregulated DEPs. The results showed that, as compared with those in the low-risk group, the content of VWF in the high-risk sera was significantly increased, while the expression of F13B did not alter significantly. This validation suggests that VWF protein may serve as a potential orthopedic surgical thrombosis biomarker (Fig. 6).

## Discussion

VTE is a complication with a high incidence rate after major orthopedic surgery, and is also one of the important factors of perioperative death and unexpected death in hospital [23, 24]. It is necessary to apply a more effective biomarker characterized by high sensitivity and specificity to guide orthopedic surgeons to prevent thrombosis. Currently, color Doppler ultrasound and D-dimer are used to evaluate the risk of thrombosis before surgery in clinical practice [25, 26], and their weakness cannot be ignored, however. Recent studies reported that some proteins such as NLR, PLR, MLR and SII in blood can be biomarkers of thrombosis, but their stability and specificity are questioned [15]. It is thus urgently needed to discover suitable biomarkers for clinicians.

Based on the well development of MS technology and proteomics, better identification, characterization and quantification of expressed proteins in plasma become a reality [27]. Here we employed the prevalent DIA-MS method to profile the proteomic alterations in sera with different coagulation risks, aiming to globally screen for potential biomarkers of thrombosis. The identified DEPs were subjected to bioinformatic analyses including KEGG pathway enrichment and chord diagram analysis to better understand the molecular pathways involved in thrombosis. ELISA experiment was then carried out to verify the significant alterations of key proteins. Our results demonstrate that plasma VWF in patients after orthopedic surgery is significantly higher than preoperative VWF and thus may be used as a biomarker of thrombosis in patients with orthopedic surgery.

Thrombosis refers to abnormal blood coagulation in the circulating blood due to certain factors or blood deposits on the inner wall of the heart or blood vessel walls [28]. However, the molecular pathways and key proteins involved in this process are largely unknown. Our KEGG pathway enrichment analysis on the plasma DEPs revealed that coagulation, hemostasis, blood coagulation, platelet degranulation, and platelet activation are the major pathways affected in thrombosis. These pathways are involved in different stages of the thrombosis process [29]. And the key proteins that play functional roles in these pathways may proceed alteration in expression level and thus become direct indicators of thrombosis.

To sort out the key proteins from the plasma DEPs, we performed chord diagram analysis and found that SAA1, VWF, FLNA, ACTB, VINC, F13B, F13A and IPSP, from the pathways of blood coagulation, humoral immune response and acute phase response were clearly associated with thrombosis. Among them, the expressions of SAA1 and VWF were significantly upregulated, while the expressions of F13B, F13A and IPSP were significantly downregulated. And our ELISA experiment verified that VWF does have a higher expression in high-risk sera and thus could serve as a thrombus biomarker in orthopedic surgery patients.

VWF (von Willebrand factor) is a major determinant of hemostasis and clot formation in arteries and veins [30]. VWF deficiency can lead to von Willebrand disease (VWD), and VWF overactivition can lead to thrombotic thrombocytopenic purpura (TTP) [31, 32]. In the process of hemostasis, VWF binds to the platelet membrane GPIb-IX complex and subendothelial collagen to mediate the adhesion of platelets at the site of vascular injury [33]. As a carrier, VWF can stabilize factor VIII via binding to factor VIII [34]. VWF and some hemostatic and fibrinolytic components are independent risk factors for thrombotic disease [35]. It has been also tested that VWF may be a marker for thrombotic risk in COVID-19 [36]. In this regard, our current finding provided new experimental evidences to support these observations. The high expression of VWF in the sera can be applied to evaluate the risk of thrombus in orthopedic patients. Due to the important function of VWF in hemostasis, further investigation on its functional mechanism may provide possibility of intervention in thrombosis and sustain its role as a potential biomarker for VTE.

## Supplementary Information

**Additional file 1.** Basic information of 26 orthopedic patients at high risk of thrombosis.

## Data Availability

The datasets used and/or analyzed during the current study are available from the corresponding author on reasonable request.

## Abbreviations

VTE: Venous thromboembolism
PE: Pulmonary embolism
DVT: Deep vein thrombosis
CTPA: Computed tomography pulmonary angiography
DIA-MS: Data-independent acquisition mass spectrometry
DEPs: Differentially expressed proteins
VWF: Von Willebrand factor
