# Supplementary material for: Identification of thrombotic biomarkers in orthopedic surgery patients by plasma proteomics
Source: J Orthop Surg Res. 2023 Mar 21;18:222. doi: 10.1186/s13018-023-03672-1 (PMC10028780; doi:10.1186/s13018-023-03672-1)
Supplement: Supplementary file 1 — Additional file 1. Basic information of 26 orthopedic patients at high risk of thrombosis. [file 13018_2023_3672_MOESM1_ESM.docx]

| Table S1: Basic information of 26 orthopedic patients at high risk of thrombosis | | | | | | | | | |
| --- | --- | --- | --- | --- | --- | --- | --- | --- | --- |
| Patient number | Age | Sex | Height（cm） | Weight（kg） | BMI | Hypertension（Yes/No） | Diabetes（Yes/No） | Diagnosis | Carprini score |
| 1 | 57 | male | 166.0 | 63.4 | 23.0 | Yes | No | Tibia comminuted fracture | 7 |
| 2 | 37 | male | 173.1 | 70.4 | 23.5 | No | No | Calcaneal fracture | 6 |
| 3 | 65 | Female | 159.8 | 57.7 | 22.6 | Yes | Yes | Intraspinal tumor | 7 |
| 4 | 68 | Female | 161.4 | 59.0 | 22.6 | Yes | No | Sacrococcygeal wound infection | 8 |
| 5 | 92 | Female | 154.0 | 50.2 | 21.2 | No | No | OVCF（T12） | 5 |
| 6 | 79 | Female | 157.2 | 52.0 | 21.0 | Yes | Yes | Osteoporosis with pathological fractures | 7 |
| 7 | 67 | male | 171.0 | 65.0 | 22.2 | No | No | After left knee arthroplasty | 7 |
| 8 | 64 | Female | 158.0 | 50.1 | 20.1 | No | No | Femoral neck fracture | 8 |
| 9 | 84 | Female | 154.7 | 47.0 | 19.6 | Yes | Yes | right femoral shaft fracture | 10 |
| 10 | 81 | Female | 159.0 | 50.9 | 20.1 | Yes | Yes | Right femoral neck fracture | 8 |
| 11 | 95 | male | 167.0 | 55.7 | 20.0 | Yes | No | Femoral neck fracture | 6 |
| 12 | 72 | Female | 161.9 | 54.6 | 20.8 | No | Yes | Fracture of left distal clavicle | 8 |
| 13 | 65 | male | 172.0 | 65.0 | 22.0 | No | No | Cervical spondylosis | 5 |
| 14 | 88 | male | 166.7 | 56.5 | 20.3 | No | No | LDH | 5 |
| 15 | 77 | Female | 168.0 | 67.0 | 23.7 | Yes | No | LDH | 5 |
| 16 | 88 | Female | 157.0 | 60.0 | 24.3 | No | No | OVCF | 5 |
| 17 | 76 | Female | 154.9 | 48.0 | 20.0 | No | No | Radius fracture | 5 |
| 18 | 47 | male | 174.0 | 65.4 | 21.6 | Yes | No | Ankle fracture | 6 |
| 19 | 62 | Female | 156.6 | 49.0 | 20.0 | Yes | Yes | Multiple strains | 7 |
| 20 | 40 | Female | 157.6 | 75.1 | 30.2 | No | Yes | Clavicle fracture | 7 |
| 21 | 66 | Female | 161.0 | 52.5 | 22.0 | No | No | Sacroiliitis | 7 |
| 22 | 50 | Female | 154.9 | 77.9 | 32.5 | No | No | LDH | 7 |
| 23 | 62 | Female | 158.6 | 51.0 | 20.3 | Yes | No | LDH | 7 |
| 24 | 73 | Female | 159.0 | 66.0 | 26.1 | Yes | No | Spondylolisthesis | 5 |
| 25 | 79 | Female | 155.0 | 53.5 | 22.3 | Yes | No | OVCF | 8 |
| 26 | 51 | Female | 165.0 | 74.0 | 27.1 | No | No | Distal left fibula fracture | 8 |
| LDH: Lumbar disc herniation; LSS: Lumbar spinal stenosis; OVCF: Osteoporotic vertebral compression fracture. | | | | | | | | | |

| Table S2: Basic information of 26 orthopedic patients at low risk of thrombosis | | | | | | | | | |
| --- | --- | --- | --- | --- | --- | --- | --- | --- | --- |
| Patient number | Age | Sex | Height（cm） | Weight（Kg） | BMI | Hypertension（Yes/No） | Diabetes（Yes/No） | Diagnosis | Carprini score |
| 1 | 34 | male | 171.0 | 62.4 | 21.3 | No | No | Back tumor | 1 |
| 2 | 49 | male | 168.5 | 66.0 | 23.2 | No | No | Femoral shaft fracture | 1 |
| 3 | 59 | male | 181.0 | 92.0 | 28.1 | Yes | No | LSS | 1 |
| 4 | 55 | Female | 152.8 | 65.2 | 27.9 | No | No | LDH | 1 |
| 5 | 47 | male | 177.0 | 72.8 | 23.2 | No | No | LDH | 1 |
| 6 | 42 | Female | 160.1 | 82.0 | 32.0 | Yes | No | LDH | 1 |
| 7 | 31 | male | 173.0 | 75.0 | 25.1 | No | Yes | Finger tendon injury | 1 |
| 8 | 27 | male | 182.0 | 82.0 | 24.6 | No | No | LDH | 0 |
| 9 | 56 | Female | 167.0 | 67.0 | 24.8 | Yes | No | Low back pain | 1 |
| 10 | 52 | Female | 155.8 | 60.0 | 24.7 | No | No | Low back pain | 1 |
| 11 | 45 | male | 173.0 | 72.0 | 24.2 | No | No | LDH | 1 |
| 12 | 24 | male | 176.2 | 83.8 | 27.0 | No | No | Knee meniscus injury | 1 |
| 13 | 46 | male | 169.0 | 77.0 | 27.1 | No | No | Cervical spondylosis | 1 |
| 14 | 46 | male | 173.0 | 61.0 | 20.3 | No | No | Cervical spondylosis | 1 |
| 15 | 55 | Female | 162.7 | 60.0 | 22.7 | No | No | Spondylolisthesis | 1 |
| 16 | 55 | male | 171.0 | 60.0 | 20.5 | No | No | LDH | 1 |
| 17 | 34 | male | 158.0 | 45.0 | 18.0 | No | No | Lower extremity pain | 0 |
| 18 | 31 | male | 171.8 | 85.6 | 29.0 | No | No | Schwannoma | 0 |
| 19 | 55 | male | 169.0 | 78.2 | 27.3 | Yes | No | LDH | 1 |
| 20 | 57 | male | 168.6 | 65.7 | 23.1 | No | No | Lumbar spinal stenosis | 1 |
| 21 | 35 | male | 173.8 | 70.0 | 23.2 | No | No | LDH | 0 |
| 22 | 69 | Female | 154.0 | 53.6 | 22.6 | No | No | Back mass | 1 |
| 23 | 34 | Female | 166.7 | 62.5 | 22.5 | No | No | Meniscus damage | 1 |
| 24 | 58 | Female | 157.9 | 72.0 | 29.2 | Yes | No | Knee meniscus injury | 1 |
| 25 | 20 | male | 176.7 | 64.0 | 20.5 | No | No | Old fracture of distal radius | 0 |
| 26 | 54 | Female | 158.0 | 57.5 | 23.0 | No | No | Double knee dysplasia | 1 |
| LDH: Lumbar disc herniation; LSS: Lumbar spinal stenosis; OVCF: Osteoporotic vertebral compression fracture. | | | | | | | | | |
